# First-principles study of half-metallicity in semi-hydrogenated BC_3_, BC_5_, BC_7_, and B-doped graphone sheets

**DOI:** 10.1186/1556-276X-6-190

**Published:** 2011-03-01

**Authors:** Yi Ding, Yanli Wang, Jun Ni, Lin Shi, Siqi Shi, Chaorong Li, Weihua Tang

**Affiliations:** 1 Department of Physics, Hangzhou Normal University, Hangzhou, Zhejiang 310036, People's Republic of China; 2 Department of Physics, Center for Optoelectronics Materials and Devices, Zhejiang Sci-Tech University, Xiasha College Park, Hangzhou, Zhejiang 310018, People's Republic of China; 3 Department of Physics, Key Laboratory of Atomic and Molecular Nanoscience (Ministry of Education), Tsinghua University, Beijing 100084, People's Republic of China; 4 Suzhou Institute of Nano-Tech and Nano-Bionics, Chinese Academy of Sciences, Suzhou 215125, People's Republic of China

## Abstract

Using first principles calculations, we investigate the electronic structures of semi-hydrogenated BC_3_, BC_5_, BC_7_, and B-doped graphone sheets. We find that all the semi-hydrogenated boron-carbon sheets exhibit half-metallic behaviors. The magnetism originates from the non-bonding *p_z _*orbitals of carbon atoms, which cause the flat bands to satisfy the Stoner criterion. On the other hand, boron atoms weaken the magnetic moments of nearby carbon atoms and act as holes doped in the sheets. It induces the down shift of the Fermi level and the half-metallicity in semi-hydrogenated sheets. Our studies demonstrate that the semi-hydrogenation is an effective route to achieve half-metallicity in the boron-carbon systems.

## Introduction

Since the discovery of graphene [1], two-dimensional (2D) nano-sheet structures have attracted lots of research in the condensed matter physics. Graphene is a monolayer carbon hexagonal sheet, in which both *α *and *β *sites of the hexagon are occupied by carbon atoms [2]. Owing to the equivalence of two carbon sites, the graphene sheet is a semi-metal with the massless Dirac-like electronic excitation [3]. When the graphene sheet connects with Si monolayer, this Dirac-like electronic structure is maintained [4]. While the graphene sheet is epitaxially grown on the SiC substrate, two carbon sites become inequivalent and a band gap is opened [5]. Recently, several chemical methods have been reported for the high-yield production of graphene [6,7]. The graphene-based transistors also develop fast, and those carbon-based nanomaterials are considered as candidates for the post-silicon electronics [8,9].

Since the prefect graphene sheet is a semi-metal with zero band gap [2], the hydrogenation is used as an effective way for the chemical functionalization of graphene [10]. The fully hydrogenated graphene sheet, called as graphane, is a semiconductor with a band gap of 3.5 eV [11-14]. In the experiments, by exposing graphene under hydrogen plasma surroundings, the graphane sheet has already been synthesized [15]. When some hydrogen atoms are removed from the graphane sheet, the magnetism will appear in those hydrogen vacancies [16]. The large area of hydrogen vacancies can even form the graphene nanoroads or quantum dots in the graphane sheets [17,18]. Under the external electric field, hydrogen atoms are pushed away from one side of the graphane sheet, while the others are still retained at the other side, which forms the semi-hydrogenated graphene sheet [19]. The previous theoretical study has shown that this semi-hydrogenated graphene, which is referred to graphone, is a ferromagnetic semiconductor with a small band gap [20]. Using the angle-resolved photoemission spectroscopy, researchers have found that the patterned one-side hydrogen adsorption can induce a band gap for the graphene sheet on the Ir (111) surface [21].

Besides the graphene sheet, the semi-hydrogenation can also tune the properties of other graphene-like 2 D sheets. For example, the semi-hydrogenated BN sheet becomes a ferromagnetic metal [22], and the semi-hydrogenated SiC sheet becomes an antiferromagnetic semiconductor [23]. By coevaporation of boron and carbon atoms, hexagonal-like boron carbides are formed with the boron content being less than 50% [24]. Moreover, the graphene-like BC_3 _sheet can be grown on the NbB_2 _(0001) surface by an epitaxial method [25]. In our previous study, we have found that the fully hydrogenation leads to the semiconductor-metal transitions in the BC_3_, BC_5_, and BC_7 _sheets [26]. Since the semi-hydrogenation can cause spin polarization in the 2 D sheets and the ordered boron-carbon compounds have rich electronic properties, the semi-hydrogenated boron-carbon sheets will be expected to exhibit interesting electronic and magnetic behaviors. It is also promising for the research on the B-doped effects on the semi-hydrogenated sheets. Thus, we perform first principles calculations to investigate the electronic structures of semi-hydrogenated BC_3 _(H-BC_3_), BC_5 _(H-BC_5_), BC_7 _(H-BC_7_), and B-doped graphone sheets in this article.

### Calculation details

First principles calculations are performed by the VASP code [27]. The approach is based on an iterative solution of the Kohn-Sham equation of the density function theory in a plane-wave set with the projector-augmented wave pseudopotentials. In our calculations, the Perdew-Burke-Ernzerhof (PBE) exchange-correlation (XC) functional of the generalized gradient approximation is adopted. We set the plane-wave cutoff energy to be 520 eV and the convergence of the force on each atom to be less than 0.01 eV/Å. The optimizations of the lattice constants and the atomic coordinates are made by the minimization of the total energy. The supercells are used to simulate the isolated sheet and the sheets are separated by larger than 12 Å to avoid interlayer interactions. The Monkhorst-Pack scheme is used for sampling the Brillouin zone. In the calculations, the structures are fully relaxed with a mesh of 5 × 5 × 1, and the mesh of **k **space is increased to 7 × 7 × 1, in the static calculations. In the spin-polarized calculations, both the ferromagnetic (FM) and antiferromagnetic (AFM) states are constructed for the initial magnetic structures of the H-BC*_x _*(*x = *3, 5, 7) sheets. However, the artificial AFM state always converges to the FM state after optimization.

## Results and discussion

**Figure 1:**
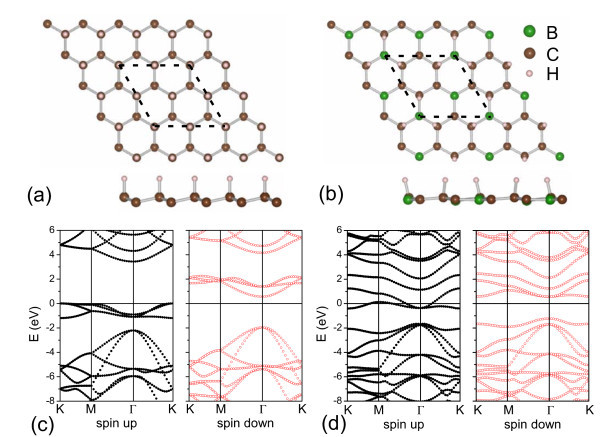
**The structures and energy bands of semi-hydrogenated sheets**. (**a**,**c**) the graphone and (**b**,**d**) the H-BC_3 _sheets. The calculated units are delineated by dotted lines in (**a**,**b**). The Fermi level is indicated as the line at *E *= 0 eV.

Figure 1 shows the structures of the graphone and H-BC_3 _sheets. In the graphone sheet, hydrogen atoms only bond with the carbon atoms at *β *sites (C*_β_*), not the carbon atoms at *α *sites (C_α_). After semi-hydrogenation, the lattice constant of graphone is increased, which is 2.75% larger than that of graphene. The calculated C-C and C-H bond lengths are 1.50 and 1.16 Å, respectively, which agree well with the previous study [20]. Owing to the inequivalence of C_α _and C*_β _*atoms, graphone is a semiconductor. As shown in Figure 1c, it has an indirect band gap of 0.48 eV, which is also in good accordance with the results by Zhou et al. [20]. In the H-BC_3 _sheet, only the C*_β _*atoms are bonding with hydrogen atoms, since under normal chemical potential, the hydrogen atoms prefer to bonding with carbon atoms in the BC_3 _sheet [26]. We have also calculated the conformation in which all the C*_β _*and B*_β _*atoms bond with hydrogen atoms. The binding energy of this conformation is - 1.40 eV/H, which is 0.13 eV/H less stable than the H-BC_3 _sheet shown in Figure 1b. The calculated B-C, C-C, and C-H bond lengths of the H-BC_3 _sheet are 1.53, 1.49, and 1.14 Å, respectively, and the lattice constant is 6.59% larger than that of graphene. Different from graphone, the C-H bonds tilt to the nearby boron atoms in the H-BC_3 _sheet. These tilting C-H bonds, together with the elongated lattice constant, decrease the repulsion between the hydrogen atoms and lead to a high binding energy of - 1.53 eV/H for the H-BC_3 _sheet.

**Figure 2:**
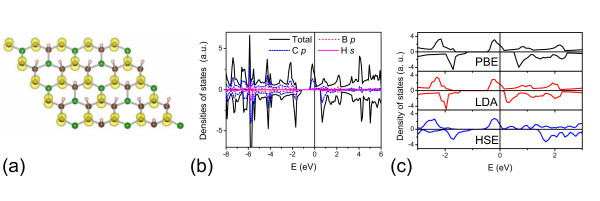
**The electronic structures of the H-BC_3 _sheet**. **(a) **The spin density distribution, **(b) **the total and partial DOSs, **(c) **the total DOS with different XC functionals of the H-BC_3 _sheet. The Fermi level is indicated as the line at *E *= 0 eV.

The band structure of the H-BC_3 _sheet is shown in Figure 1d. Different from the semiconducting graphone, the H-BC_3 _sheet exhibits a half-metallic character. There are two at bands crossing the Fermi level for the spin-up electrons. On the other hand, for the spin-down electrons, it opens a band gap of 1.76 eV. The half-metal gap, defined as the difference between the Fermi level and topmost occupied spin-down band, is 1.18 eV for the H-BC_3 _sheet. We have also checked the half-metallicity of the H-BC_3 _sheet with different XC functionals. Figure 2 displays the calculated densities of states (DOSs) by the Ceperly-Alder functional form of the local density approximation and the hybrid XC functional of Heyd-Scuseria-Ernzerhof. Both calculations confirm the half-metallic behavior of the H-BC_3 _sheet.

In order to gain more insight into the half-metallicity, we plot the spin density distribution and partial DOSs of the H-BC_3 _sheet as shown in Figure 2. The figure indicates that the magnetism is mainly from the *p_z _*orbitals of C_α _atoms. The C_α _atom is not hydrogenated in the H-BC_3 _sheet. It has an unpaired *p*-electron localized in the non-bonding *p_z _*orbital, which contributes to the flat bands near the Fermi level. The at bands lead to large DOSs flat the Fermi level, which are beneficial to satisfy the Stoner criterion, *IN*(*E*_F_) > 1 and induce the ferromagnetism in the semi-hydrogenated sheet [28]. For the graphone sheet, there are also flat bands near the Fermi level as shown in Figure 1c, which cause spin polarization of those unhydrogenated C_α _atoms [20]. However, owing to the existence of boron atoms, the magnetism of H-BC_3 _sheet is weakened. For the same calculated units in Figure 1, the graphone sheet has a total magnetic moment of 4*μ_B_*, while the H-BC_3 _sheet has only 1*μ_B_*. Using the Bader analysis [29], we obtain that the boron atom transfers 1.27 *e *to the surrounding C_α _atoms. Each C_α _atom contributes 0.79*μ_B _*in the graphone sheet, while in the H-BC_3 _sheet it decreases to 0.31*μ_B _*because of the charge transfers from nearby boron atoms. Considering that the boron element is one electron less than the carbon one, the boron atoms behave like holes doped in the semi-hydrogenated sheets. It leads to the down shift of the Fermi level, which crosses the spin-up bands. Consequently, the H-BC_3 _sheet becomes a half-metal.

**Figure 3:**
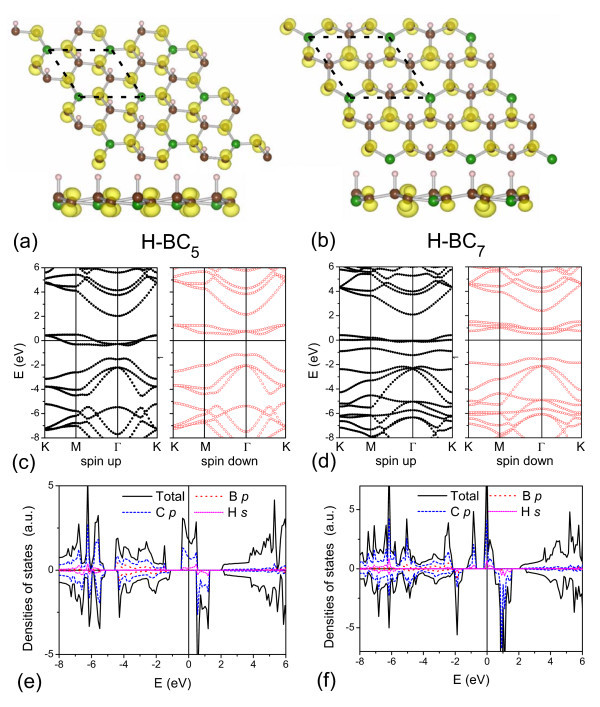
**The electronic structures of the H-BC_5 _and H-BC_7 _sheets**. (Color online) The structures, energy bands, and DOSs of **(a,c,e) **the H-BC_5 _and **(b,d,f) **the H-BC_7 _sheets. The calculated units are delineated by dotted lines, and the spin density distributions are shown in **(a,b)**. The Fermi level is indicated as the line at *E *= 0 eV.

**Figure 4:**
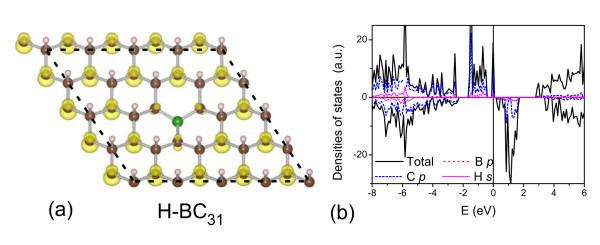
**The electronic structures of the B-doped graphone sheet**. (Color online) **(a) **The structures and **(b) **DOSs of the B-doped graphone sheets. The calculated units are delineated by dotted lines and the spin density distributions are shown in **(a)**. The Fermi level is indicated as the line at *E *= 0 eV.

More interestingly, the half-metallicity appears not only in the H-BC_3 _sheet, but also in other semi-hydrogenated boron-carbon sheets. Figure 3 shows the electronic structures of the H-BC_5 _and H-BC_7 _sheets. The magnetism is also mainly localized at the C_α _atoms of those sheets. In the H-BC_5 _sheet, the C_α _atom has a magnetic moment of 0.31*μ_B_*. On the other hand, in the H-BC_7 _sheet, the atomic magnetic moments become 0.34 and 0.72*μ_B_*. The two values correspond, respectively, to the C_α _atoms with and without neighboring boron atoms. Both the H-BC_5 _and H-BC_7 _sheets are half-metals, the half-metal gaps of which are 1.12 and 1.50 eV, respectively. To model the B-doped graphone sheet, one C atom is replaced by the B atom in a 4 × 4 unit cell, yielding a B-doped concentration of 3.125%. Figure 4a displays that the doped boron atom weakens the magnetism of three neighboring C_α _atoms. Comparing with the prefect graphone sheet, the total magnetic moment is reduced by 2*μ_B _*after boron doping. The B-doped graphone sheet also presents a half-metallic behavior as shown in Figure 4b.

Table 1 listed the calculated results. All the semi-hydrogenated boron-carbon sheets are half-metals. We find that the different boron contents have two effects on the stabilities of half-metallic sheets: on the one hand, with the increase of the boron contents, the binding energies increase because of the decreased repulsion between hydrogen atoms with the elongated lattice constants. On the other hand, the boron atoms weaken the nearby C_α _magnetic moments, which decreases the *p*-*p *interactions between them. Thus, the energy gain of the ferromagnetic state decreases with the increase of the boron contents. Comparing with the normal room temperature (25 meV), the half-metallicities of the H-BC_3_, H-BC_5_, and H-BC_7 _sheets are still stable.

**Table 1:** The binding energy $E_{\text{b}}=E_{\text{H}-\text{BC}}{}_{{}_{\text{X}}}-E_{{\text{BC}}_{\text{X}}}-E_{\text{Hatom}}$, the increasing rate of lattice constant relative to grapheme ϵ_rare _= (*a*-*a*_Graphene_)/(*a*_Graphene_), the energy gain of the ferromagnetic state *E*_M _= *E*_FM _- *E*_NM_, the total magnetic moment *m*_total_, the carbon atomic magnetic moment $m_{C_{\alpha }}$, and the electronic property for each semi-hydrogenated sheet

	*E*_b_(eV/H)	*ε*_rate _(%)	*E*_M_(meV/C*_a_*)	*m*_total _*(μ_B_*)	$m_{C_{\alpha }}$ (*μ_B_*)	Property
Graphone	-0.63	2.75	-271	4.00	0.79	Semiconductor (0.4 eV)
H-BC_3_	-1.53	6.59	-51	1.00	0.31	Half-metal (1.18 eV)
H-BC_5_	-1.10	4.47	-47	1.00	0.31	Half-metal (1.12 eV)
H-BC_7_	-0.94	4.11	-84	2.00	0.34/0.72	Half-metal (1.50 eV)
B-doped graphone	-0.67	2.96	-221	14.00	0.35/0.78	Half-metal (2.25 eV)

The numerical values are listed following "Semiconductor" is the band gap, and those listed following "Half-metal" are half-metal gaps

## Conclusions

In summary, we find that all the semi-hydrogenated BC_3_, BC_5_, BC_7_, and B-doped graphone sheets are half-metals. The magnetism originates from the non-bonding *p_z _*orbitals of C_α _atoms. The boron atoms weaken the nearby C_α _magnetic moments, and cause the Fermi level to shift into the spin-up states. A half-metal gap is opened in the spin-down bands, the value of which is about 1-2 eV depending on the boron contents. Owing to the promising half-metallicity, the semi-hydrogenated boron-carbon sheets have potential applications in spintronics and nanodevices.

## Abbreviations

AFM: antiferromagnetic; DoSs: densities of states; FM: ferromagnetic; PBE: Perdew-Burke-Ernzerhof.

## Competing interests

The authors declare that they have no competing interests.

## Authors contributions

YD and YW conceived the idea, performed the calculations, analyzed the data, and wrote the manuscript. JN, LS, SS, CL, and WT participated in the study. All authors read and approved the final manuscript.
